# Assessment of erectile dysfunction prevalence and associated factors in hypertensive men

**DOI:** 10.7717/peerj.18596

**Published:** 2024-11-27

**Authors:** Mahruk Rashidi, Neşe Kıskaç, Deniz Kaya Meral, Sultan Çakmak, Ebru Durusoy, Aydın Nart, Dilara Cengizli, Esra Özer, Meltem Aslan, Muharrem Kıskaç

**Affiliations:** 1 Department of Nursing/Faculty of Health Sciences, İstanbul Gelişim University, İstanbul, Turkey; 2 Department of Physiotherapy and Rehabilitation/Faculty of Health Sciences, İstanbul Gelişim University, İstanbul, Turkey; 3 Department of Internal Medicine, Bezmialem Vakıf University, İstanbul, Turkey

**Keywords:** Hypertension, Erectile dysfunction, Risk factors, Multidisciplinary approach

## Abstract

**Background:**

This study aimed to assess the prevalence of erectile dysfunction and identify associated factors among male patients with hypertension.

**Methods:**

A cross-sectional descriptive study was conducted. Data were gathered from 223 individuals aged 18 and above, solely diagnosed with hypertension. Information was collected through face-to-face questionnaires, personal identification forms, and the 5-question version of the International Sexual Function Index Scale.

**Results:**

Among the participants, 81.6% exhibited erectile dysfunction, with a mean total score of 18.72 ± 3.60 on the 5-question version of the International Index of Erectile Function Questionnaire, indicating a mild level of dysfunction. Older age, smoking, lower educational attainment, and use of beta blockers were associated with higher levels of erectile dysfunction (*p* < 0.05).

**Discussion:**

To mitigate modifiable risk factors influencing erectile dysfunction severity in hypertensive males, promoting healthy lifestyle choices, including diet, exercise, physiotherapy, and psychosocial support, as well as educating patients and their partners, could prove beneficial as non-pharmacological interventions.

## Introduction

Hypertension is a major health problem, affecting more than 1.1 billion people worldwide and causing significant vascular damage (Tsoutsos & Kotsis, 2023). Cardiovascular diseases and organ dysfunctions develop in the later stages of hypertension. Therefore, it is very important to detect hypertension-related endothelial dysfunction and thus the atherosclerotic process at an early stage to prevent complications that may develop (Karakurt et al., 2020). One of these complications is erectile dysfunction (ED). Erectile dysfunction is defined as the inability to achieve and/or maintain an erection continuously for at least 3 months for a satisfactory sexual intercourse. The prevalence in the general population is reported to be 50% after the age of 40 (De Oliveira & Nunes, 2021). However, it is estimated that 322 million men will be affected by this disease by 2025 (Kessler et al., 2019; Yafi et al., 2016; Cantone et al., 2022). ED not only affects sexual life in men, but also negatively affects the social, economic and psychological life of the individual (Ates et al., 2020; Karakurt et al., 2020). The etiology of erectile dysfunction involves multifactorial factors such as hypertension, atherosclerosis, diabetes, decreased physical activity, heart disease, smoking and polypharmacy (Wang et al., 2021; Teixeira & Soares, 2020; Tsoutsos & Kotsis, 2023). Endothelial dysfunction caused by hypertension can lead to erectile dysfunction if atherosclerotic vasculature occurs in the penile artery (Karakurt et al., 2020).

This condition, caused by hypertension, often poses a risk to patients when it goes unrecognized and therefore untreated (Corradetti et al., 2022). In this situation, both hypertension needs to be managed effectively and ED needs to be addressed with appropriate treatment options. While several classes of antihypertensive drugs, including mineralocorticoid receptor antagonists, centrally acting drugs, *β*-blockers and diuretics, commonly cause erectile dysfunction (Altunören et al., 2019; Manolis et al., 2020), ED is also reported to be high in male patients with risky lifestyles such as sedentary lifestyle, diet, obesity, smoking habit and high alcohol consumption (Hernández-Cerda et al., 2020; Rezali et al., 2023).

Smoking cessation, weight loss and maintenance, regular physical exercise (especially aerobic exercise), stress management, reduction of salt and alcohol consumption, and dietary changes (Mediterranean diet) are common lifestyle change measures to reduce hypertension, the risk of ED and the risk of hypertension-related cardiovascular complications (Carella et al., 2023). Therefore, it is very important to evaluate and treat ED, which has many risk factors, from a multidisciplinary perspective.

Understanding the extent of ED and the contributing risk factors among male patients with hypertension is crucial for developing effective intervention strategies. Thus, this study aims to assess the prevalence of ED and its associated factors in hypertensive male patients.

**Research questions:**

1. What is the level of erectile dysfunction in hypertensive men?

2. What is the relationship between personal data and erectile dysfunction in hypertensive men?

3. What is the relationship between medications used by hypertensive men and erectile dysfunction?

## Materials and Methods

### Design

The study was conducted using a cross-sectional and descriptive design. The sample of the study consisted of 223 voluntary patients aged 18 years and over who applied to the cardiology outpatient clinic and had no other medical diagnosis other than hypertension. Data were collected *via* face-to-face surveys conducted at outpatient cardiolology clinics of a public hospital, a city hospital, a training and research hospital, and a private hospital between November 10, 2023, and March 01, 2024.

### Data collection tools

The study utilized the 5-item version of the International Index of Erectile Function (IIEF-5) questionnaire along with a personal data identification form. The personal data identification form included demographic information such as gender, age, marital status, education level, years of hypertension, current medications, smoking status, years of smoking, and alcohol use.

#### Five-item version of the International Index of Erectile Function (IIEF-5)

The IIEF Form, developed in 1997 for assessing sexual functions in men (Jose & Alfons, 2007), has been widely recognized for its validity, particularly in large-scale multicenter clinical studies. IIEF was translated into Turkish by the “Turkish Andrology Association” (Aydin, 1998). Scores on the scale range from a minimum of 5 to a maximum of 25. Interpretation of scores categorizes 5–7 points as severe, 8-11 points as moderate, 12–16 points as mild-moderate, 17–21 points as mild, and 22–25 points as indicating no ED.

### Data analysis

The data analysis for the study was conducted using IBM SPSS 26.0 statistical software (IBM, Armonk, NY, USA). Descriptive statistics such as mean, standard deviation, frequency, and percentage were calculated. Student’s *t*-test was utilized for comparing normally distributed data, while the Mann–Whitney U test was employed for non-normally distributed data. One-way ANOVA and Kruskal-Wallis tests were applied to assess variables with more than two normal and non-normal distributions, respectively. Correlation analyses, including Pearson and Spearman correlation, were conducted to examine the relationships between variables. The significance level was set at *p* < 0.05, and results were evaluated based on a 95% confidence interval.

### Ethical Considerations

The study obtained ethical approval from the Bezmialem Vakıf University Non-Interventional Research Ethics Committee, with decision number 2023/145, dated June 14, 2023. Before completing the questionnaire, participants were informed about the study, and written consent forms were obtained from each participant.

## Results

Table 1 presents the personal data of the participants along with the total score of IIEF. The participants had a mean age of 63.26 ± 7.62 years, with 97.3% being married. Additionally, 46.6% of participants were primary school graduates, 9.4% reported alcohol use, and 13% were smokers, with an average duration of smoking of 22.83 ± 6.94 years. The mean duration of hypertension was 7.92 ± 4.06 years, and the most commonly used medication among participants (27.8%) was ACE Inhibitors. Notably, 81.6% of participants had erectile dysfunction, with the mean IIEF total score indicating a mild level of dysfunction (18.72 ± 3.60) (Table 1).

**Table 1:** Participants’ personal data, IIEF total score (*n* = 223).

	**n**		**%**
**Age (years)**		63.26 ± 7.62	
**Marital status**			
Single	6		2.7
Married	217		97.3
**Smoking**			
Yes	29		13.0
No	194		87.0
**Duration of smoking**		22.83 ± 6.94	
**Alcohol use**			
Yes	21		9.4
No	202		90.6
**Duration of hypertension**		7.92 ± 4.06	
**Educational level**			
Literate	12		5.4
Primary school graduate	104		46.6
High school graduate	99		44.4
University graduate	8		3.6
**Medicines used**			
ACE Inhibitors	62		27.8
Angiotensin Receptor Blockers	52		23.3
Beta Blockers	48		21.5
Diuretics	27		12.1
Calcium Channel Blockers	34		15.2
**Presence of erectile dysfunction**			
Yes	182		81.6
No	41		18.4
**IIEF (mean)**		18.72 ± 3.60 (min 5-max 25 puan)	
Descriptive statistical methods (mean, standard deviation, frequency, percentage)			

Table 2 illustrates the relationship between the personal characteristics of the individuals and the IIEF total score. Significant correlations were found between age, smoking status, education level, medications used, and the IIEF score (*p* < 0.05). Specifically, as age increased, the prevalence of ED was higher. Moreover, smokers exhibited a greater likelihood of experiencing ED, while individuals with lower education levels also showed a higher prevalence of ED. Additionally, beta blocker users demonstrated a significant association with ED (*p* < 0.05).

**Table 2:** Relationship between participants’ personal characteristics and IIEF total score (*n* = 223).

	**IIEF total scores**	***p*-value**
**Marital status**		
Married	18.68 ± 3.58	0.268
Single	20.33 ± 4.41	
**Age**		
≥63	18.14 ± 3.71	0.011
<63	19.37 ± 3.38	
**Smoking (Tobacco)**		
Yes	15.31 ± 2.79	0.001
No	19.23 ± 3.44	
**Duration of smoking**		
≥22	15.87 ± 2.85	0.274
<22	14.71 ± 2.70	
**Alcohol use**		
Yes	19.86 ± 3.24	0.130
No	18.60 ± 3.63	
**Educational level**		
Literate	15.75 ± 4.57	0.009
Primary school graduate	18.61 ± 3.59	
High school graduate	19.29 ± 3.33	
University graduate	17.63 ± 3.58	
**Medicines used**		
ACE Inhibitors	18.53 ± 3.95	0.001
Angiotensin Receptor Blockers	19.90 ± 2.97	
Beta Blockers	15.98 ± 3.00	
Diuretics	19.67 ± 3.45	
Calcium Channel Blockers	20.38 ± 2.43	
Descriptive statistical methods (mean, standard deviation), Student’s *t*-test, Mann–Whitney U test, ANOVA and Kruskal-Wallis test		

Table 3 displays the correlation between age, duration of hypertension, duration of smoking, and the IIEF total score. Significant negative correlations were observed between age and the IIEF total score, as well as between the duration of hypertension and the IIEF total score (*p* < 0.05). These findings indicate that as age and the duration of hypertension increase, the severity of ED tends to worsen.

**Table 3:** Correlation between age, duration of hypertension, duration of smoking and IIEF total.

		**Age**	**Duration of hypertension**	**Duration of smoking**	**IIEF**
**Age**	**r**	1	0.597	0.441	−0.333
	***p***		0.001	0.017	0.001
**Duration of hypertension**	**r**	0.597	1	0.152	−0.240
	***p***	0.001		0.432	0.001
**Duration of smoking**	**r**	0.441	0.152	1	0.025
	***p***	0.017	0.432		0.898
**IIEF**	**r**	−0.333	−0.240	0.025	1
	***p***	0.001	0.001	0.898	
Pearson and Spearman correlation analysis					

## Discussion

In this study, the focus was on understanding the prevalence of ED among hypertensive patients and exploring the relationship between ED and the patients’ personal characteristics. ED is a prevalent health issue that significantly impacts a man’s mental health, relationships, and overall well-being, thereby affecting quality of life (Mitidieri et al., 2020). Therefore, addressing various factors impacting the general health of men with ED is crucial.

Given that erectile physiology is largely influenced by vascular changes, cardiovascular risk factors like hypertension can hasten the onset of ED. Studies have indicated that the prevalence of ED in hypertensive patients increases with age (Ferrini, Gonzalez-Cadavid & Rajfer, 2017; Irfan et al., 2020). For instance, Pellegrino et al. (2023) reported that the likelihood of ED in individuals aged 50 to 75 years was between 20% and 68% in healthy men and between 41% and 85% in those with hypertension, obesity, and diabetes. Similarly, Boombhi et al. (2019) highlighted the influence of the duration of hypertension on ED. Consistent with existing literature, age and the duration of hypertension were found to be significant factors affecting ED in this study. As individuals age, their cardiovascular health may deteriorate, leading to the development of chronic diseases and associated complications.

Smoking poses serious risks to all organ systems, with approximately one-third of men worldwide being active smokers (World Health Organization, 2018). Lifestyle habits such as smoking, alcohol consumption, and substance use have been identified as risk factors for ED (Mazzilli, 2022; Sivaratnam et al., 2021). Numerous studies have underscored the detrimental effects of smoking on erectile function (Allen & Tostes, 2023; Corona et al., 2020). Similarly, in this study, smoking was found to have a significant impact on ED. Cigarette smoke contains nicotine, carbon monoxide, oxidant chemicals and metals that can damage the endothelium and impair erection processes (Allen & Tostes, 2023). Therefore, smoking is an important risk factor for ED and cardiovascular diseases as it causes endothelial dysfunction, oxidative stress and inflammation (Carella et al., 2023). Smoking can lead to vasoconstriction and reduced blood flow, impairing the ability to achieve or sustain an erection. Additionally, smoking can contribute to the buildup of plaque on vessel walls, leading to arteriosclerosis. This, in turn, can affect penile blood flow and result in erection difficulties. Furthermore, smoking can disrupt the nervous system and hormonal balance, further exacerbating erectile dysfunction. The chemicals present in cigarettes are believed to affect the nervous system and hormones, thereby diminishing sexual performance.

The results of this study revealed that the prevalence of ED increased as the educational level of the individuals decreased. Additionally, the analysis showed that the mean age increased as the educational level decreased, with literate individuals having a higher mean age compared to university graduates (mean age 71.33 ± 12.30 for literate individuals and 58.25 ± 4.33 for university graduates). Moreover, it was observed that the prevalence of ED increased as the mean age increased. Although educational level does not directly influence the frequency of ED, it is believed to have an indirect impact.

*β*-blockers represent one of the primary classes of antihypertensive drugs recommended for hypertension treatment (Williams et al., 2018). In studies investigating the effects of *β*-blockers on ED, negative effects of this drug class have been reported (Hernández-Cerda et al., 2020; Manolis et al., 2020; Terentes-Printzios et al., 2022). Consistently, in this study, higher levels of ED were observed in patients using *β*-blockers. This finding is consistent with existing literature on the subject.

## Limitations

The study has some limitations. Research on erectile dysfunction is limited because sexuality is culturally regarded as taboo in Turkey, making it difficult for individuals to openly express their problems and evaluate treatment options. Therefore, it is recommended that the study be developed with a larger, multicenter sample group.

## Conclusion

Erectile dysfunction represents a significant health concern that can manifest in male patients with hypertension. Hypertensive men should be regularly evaluated for sexual dysfunction, particularly erectile dysfunction. Patients should be informed about this potential side effect and encouraged to report any changes in functioning to their doctor. In this study, age, smoking, educational status, year of hypertension and *β*-blockers used as antihypertensives were found to be factors that increased the prevalence. Considering the modifiable risk factors that increase ED in hypertensive men, it is important to adopt non-pharmacologic treatment approaches such as diet, exercise, physiotherapy, psychosocial interventions, patient and partner education before starting pharmacologic treatment by encouraging individuals to lead a healthy lifestyle. Pharmacologic treatment strategies include reducing the dose of medication, switching to another class of medication or adding another agent. Treatment of sexual dysfunction will help improve medication adherence in the treatment of hypertension. In addition, the physical and mental health of hypertensive men with ED, which may negatively affect the quality of life of hypertensive men, should be evaluated with a multidisciplinary team and perspective, and care and treatment should be organized.

##  Supplemental Information

10.7717/peerj.18596/supp-1 Data S1 Raw data

10.7717/peerj.18596/supp-2 Supplemental Information 2 Strobe checklist

## Additional Information and Declarations

### Data Availability

The following information was supplied regarding data availability: The raw data are available in the Supplemental File.
